# National Nutrition and Physical Activity Surveillance in Papua New Guinea: data showing comparison with three Melanesian countries

**DOI:** 10.1016/j.dib.2026.113170

**Published:** 2026-08-12

**Authors:** Takashi Nakagata, Stewart Sarieng Pau, Vicky Wari, Masayo Rossignoli Nakamori, Rei Ono, Miwa Yamaguchi

**Affiliations:** a Center for Physical Activity Research, National Institutes of Biomedical Innovation, Health and Nutrition, NODE Lab Kento, 3-17 Senrioka Shinmachi, Settsu-shi, Osaka 566-0002, Japan; b Disease Surveillance and Rapid Response, Morobe Provincial Health Authority, ANGAU Hospital, P.O. Box 457, Lae 411, Morobe, Papua New Guinea; c Non-Communicable Diseases Branch, Diseases Control Division, Public Health Wing, National Department of Health, AOPI Centre, Waigani Drive, P O Box 807, Waigani 121, Port Moresby, Papua New Guinea; d International Nutrition Collaboration, Center for Private-Public-Academic Collaboration Research, National Institutes of Biomedical Innovation, Health and Nutrition, NODE Lab Kento, 3-17 Senrioka Shinmachi, Settsu-shi, Osaka 566-0002, Japan; e College of Gastronomy Management, Ritsumeikan University, 1-1-1 Noji-higashi, Kusatsu, Shiga 525-8577, Japan

**Keywords:** National nutrition survey, Physical activity, Non-communicable diseases, Comparison, Papua new guinea

## Abstract

This data article summarizes key health-related indicators collected through national surveys conducted in four Melanesian countries: Papua New Guinea, the Solomon Islands, Vanuatu, and Fiji. The dataset includes variables on nutrition, physical activity, anthropometric measurements, and the frequency of survey implementation. The data were compiled from publicly accessible sources, including national statistics offices, the World Health Organization (WHO), the World Bank, Asia Pacific Report, and UNESCO. The dataset for each country details the survey items, frequency of implementation, population coverage (by age and gender), data availability status, and the most recently recorded values. In addition, indicators related to physical activity and vegetable consumption were extracted from the WHO STEPwise approach to non-communicable disease (NCD) surveillance, enabling cross-country comparisons based on standardized methodologies. This dataset supports future cross-national analyses and may be valuable for public health planning, health system monitoring, and program evaluation. It offers a foundation for examining regional health patterns, particularly in relation to nutrition and NCD surveillance, in the Pacific region. The dataset is available in Mendeley Data, with detailed supplementary tables provided to encourage reuse and transparency.

Specifications Table

**Table:** 

Subject	Health Sciences, Medical Sciences & Pharmacology
Specific subject area	*National Nutrition and Physical Activity Surveillance in Papua New Guinea*
Type of data	*Tables, Figures*
Data collection	*Publicly available secondary data were collected from online repositories maintained by official sources, including national statistical offices, the World Health Organization (WHO), the World Bank, and peer-reviewed publications.*
Data source location	*The data were retrieved from publicly accessible online sources including:* *World Health Organization* (https://extranet.who.int/ncdsmicrodata/index.php/catalog/STEPS/?page=1&ps=15&repo=STEPS) *The World Bank Open Data* (https://data.worldbank.org/) *National Statistical Offices of Papua New Guinea, the Solomon Islands, Vanuatu, and Fiji*
Data accessibility	Repository: Mendeley Data Direct link to the data: https://data.mendeley.com/datasets/fx82bcnf8v/3 Persistent identifier of the data: 10.17632/fx82bcnf8v.3
Related research article	*None*

## 1 Value of the Data

• This dataset brings together, in a standardized format, key indicators from national surveys in four Melanesian countries (Papua New Guinea, Solomon Islands, Vanuatu, and Fiji). To our knowledge, few publicly accessible resources have compiled these heterogeneous data sources into a single structure, improving accessibility for researchers working on regional health surveillance.
• The dataset includes information on survey frequency, target populations, sampling frames, and measurement domains. These metadata can help researchers describe the current status of health monitoring systems in Melanesia and may support future discussions on survey design or implementation.
• By organizing indicators from both National Nutrition Surveys and WHO STEPS, the dataset provides structured access to variables related to nutrition, physical activity, and anthropometry. Researchers can reuse these variables to examine patterns across countries or to construct country profiles based on harmonized definitions.
• Because the dataset documents the timing, scope, and measurement approaches of existing surveys, it may be informative for researchers or agencies when considering which survey items or age groups have historically been included in national surveillance systems.
• The unified format may facilitate methodological comparisons across countries, such as examining similarities or differences in measurement approaches, target ages, or indicator definitions. These comparisons can support future work on harmonizing surveillance systems in resource-constrained settings.

## 2 Background

Papua New Guinea (PNG), a Melanesian country in Oceania, shares a range of public health challenges with its neighboring nations, particularly in relation to nutrition and physical activity [1]. As non-communicable diseases (NCDs) continue to rise globally and within the region, timely and reliable information from national surveillance systems is essential for public health assessment and planning. In this context, understanding how national surveys are implemented—including their frequency, scope, and accessibility—is important for interpreting existing data and guiding future monitoring efforts.

This study provides a structured description of the publicly available national surveillance data in PNG, focusing on the National Nutrition Survey (NNS) [2] and the WHO STEPwise approach to surveillance (STEPS) for physical activity and related indicators [3,4]. The aim is to summarize the current characteristics of these surveys and to document how PNG's surveillance landscape aligns with that of other Melanesian countries. The results are intended to support the development of complementary linkages among Melanesian countries in order to strengthen national surveys.

For contextual comparison, three neighboring countries—the Solomon Islands, Vanuatu, and Fiji—were included. We conducted a descriptive examination of their available national surveys, emphasizing survey implementation history, indicator coverage, data accessibility, and target population characteristics. The purpose of this comparison is not to draw analytical conclusions but to provide an overview of the surveillance systems in the Melanesian region and to highlight opportunities for strengthening future data collection efforts.

## 3 Data Description

### 3.1 PNG national nutrition survey (NNS)

The dataset compiled for this article includes the key indicators reported in the 2005 NNS, such as anthropometry, micronutrient status, and household characteristics, alongside metadata describing survey design and implementation. The NNS was designed to demonstrate the nutritional status of a population and obtain basic data to support the comprehensive promotion of public health, ranging from policies and planning to implementation and assessment. The first NNS in PNG was conducted in 1982–1983 [5]. Based on the findings of this survey, the PNG National Food and Nutrition Policy (originally formulated in 1978) was revised and developed into the 1995 National Nutrition Policy (NNP). The second NNS, conducted in 2005 after a 22-year interval, reviewed earlier findings and provided valuable insights into the persistent nutrition challenges in the country. It highlighted the triple burden of malnutrition, including undernutrition, overnutrition, and micronutrient deficiencies, which remain significant public health issues affecting people across the lifecycle. This dataset emphasizes the evolution of nutrition and physical activity policies in PNG, elucidating how these policies have been developed and adapted over time to address ongoing nutrition issues.

Table 1 provides an overview of the most recent NNS in PNG (2005), together with comparable indicators from three neighboring Melanesian countries in the Oceania region. The survey was conducted only twice in PNG (1982–1983 and 2005), which is characterized by infrequent implementation. The most recently available data are from 2005, indicating that more up-to-date data are required. In contrast, Fiji conducted the survey four times (1982, 1993, 2004, and 2015), demonstrating a higher frequency of implementation than PNG. Supplementary Table 1 presents additional information on geographic, demographic, and socioeconomic characteristics of the four Melanesian countries.

**Table 1:** Summary of the current national nutrition survey for papua new guinea compared with three melanesian neighboring countries in the oceania region.

Countries, Year	Target selection	Population in the current survey	Data availability ^a^		Years of historical implementation of the survey
			Statistical data	Individual data	
Papua New Guinea, 2005	Randomized selection from each four region	Household (HHs): 1403	Need approval	Need approval	1982–1983, 2005
	1 region Southern: 342 HHs	Men: 804			
	2 region Highlands: 359 HHs	Women: 847			
	3 region Mamose: 354 HHs	Children: 937			
	4 region Islands: 348 HHs				
Solomon Islands, 1990	Not available	Not available	Need approval	Need approval	1970, 1990
Vanuatu, 1996	Not available	Not available	Need approval	Need approval	1983, 1996
Fiji, 2015	Randomized selection (1% of the total population)	Household: 1935	Need approval	Need approval	1982, 1993, 2004, 2015
		Individuals: 6185			

Accessing data is required through an approval.

HHs; households.

**Fig. 1:**
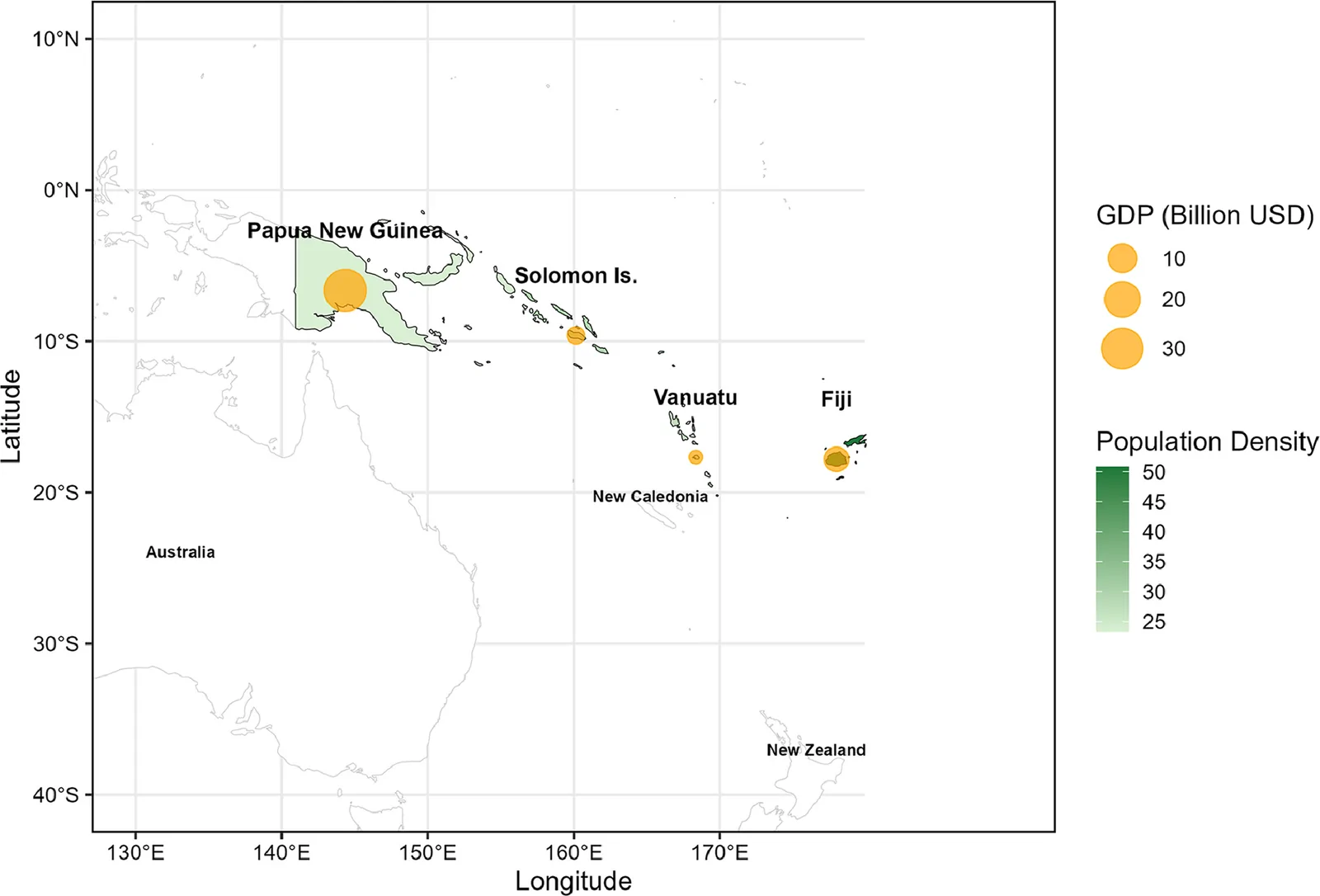
Melanesian countries with population density and GDP visualization.

Fig. 1 presents a descriptive visualization of population density and gross domestic product (GDP) for the four Melanesian countries included in this study. The map was created using the ggplot2 and sf packages in R. Geographic data were obtained and processed using the rnaturalearth and rnaturalearthdata packages.

### 3.2 WHO steps survey

In this data article, STEPS Fact Sheets were used to summarize physical activity and fruit and vegetable intake in PNG, the Solomon Islands, Vanuatu, and Fiji [3,6]. The WHO STEPS survey is a standardized method developed by the WHO to monitor the risk factors associated with non-communicable diseases (NCDs)[3]. This globally implemented approach is based on the principle that small-scale, high-quality data are more valuable than large volumes of low-quality data. The data collected through WHO STEPS play a crucial role in prioritizing NCD-related interventions, informing policy development, and evaluating the effectiveness of public health interventions at the national level.

Table 2 presents an outline of the WHO STEPS survey for PNG compared with three neighboring Melanesian countries in the Oceania region, using the most recent data available for each country.

**Table 2:** Outline of the WHO STEPS survey for papua new guinea compared with three melanesian neighboring countries in the oceania region — latest survey year only.

Country	Papua New Guinea	Solomon	Vanuatu	Fiji
Year	2007–2008	2015	2011	2011
Geographic coverage	Four regions of PNG and Port Moresby (capital only) a	National coverage	National coverage	National coverage
Universe	Adults aged 15–64 years.	Adults aged 18–69 years.	Adults aged 25–64 years.	Adults aged 25–64 years.
Sampling procedure	The data are based on a representative population-wide sample of Papua New Guineans aged 15–64 years. Representative areas in the four regions of PNG, as well as in the capital Port Moresby	A multi-stage A multi-stage cluster sample design was used to produce representative data.	A multi-stage cluster sampling design was used to produce representative data for that age range in Vanuatu.	A multi cluster stage sample design to produce representative data for adults aged 25–64 years.
Response rate	80.2%	58.4%	94%	53.3% b
Sample size	A total of 2944 individuals aged 15–64 yrs.	A total of 2525 individuals aged 18–69 yrs.	A total of 4649 individuals aged 25–64 yrs.	A total of 2586 individuals aged 25–64 yrs.
Dietary intake measurements	Dietary assessments were made by brief dietary assessment instruments: a retrospective method in which a limited number of specified food and drinks (Fruits and vegetables) were usually consumed. Mean number of days they were consumed in a typical week and mean number of servings on average per day was asked as a semi-quantitative instrument. The questionnaire was interview-administered (face-to-face).	Food items were added for rice and noodles, bread and bakery products, and sugar-added drinks. For dietary salt, respondents were asked about the amount and frequency of salt or salty sauces added, the amount of salty processed food consumed, their knowledge of salt and its impact on health, and the actions they have taken to control salt intake. For oil and fat used in meal preparation, respondents were additionally asked about the frequency of eating out and the frequency of meals containing coconut, cream or lolo.	The same as the survey conducted in Papua New Guinea in 2007–2008.	The type of oil or fat used in food preparation at household was additionally asked.
Physical activity measurements	Survey participants were asked how often (frequency) and how long (duration) they engaged in three domains of physical activity: during recreation or leisure time, work and transport in a typical week. In the work and leisure domains, respondents were asked how many days per week and how many hours/minutes per day they participate in moderate- and vigorous-intensity activities. In the transport domain, respondents were asked how often and how long they either walk and/or cycle to and from places.	A population’s physical activity (or inactivity) can be described in different ways. The two most common ways used for analysing Global Physical Activity Questionnaire (GPAQ) data are: 1) To estimate a population’s mean or median physical activity using a continuous indicator such as MET-minutes per week or time spent in physical activity. 2) To classify the population into specific groups by setting cut-off points for a specific amount of physical activity.	Respondents were asked how often (frequency) and how long (duration) they engaged in three domains of physical activity in a typical week: work-related, transport-related and leisure-related. In the work and leisure domains, respondents were asked how many days per week and how many hours/minutes per day they participate in moderate and vigorous intensity activities. In the transport domain, respondents were asked how often and how long they either walk and/or cycle to and from places.	Unknown

https://extranet.who.int/ncdsmicrodata/index.php/catalog/STEPS/?page=1&ps=15&repo=STEPS.

a For the Papuan sample, the villages of Lese Havihara and Lese Kavora in Gulf Province were selected as representative. For Momase, Ambana and Mambuan villages in Bogia District of Madang Province were studied. In the Highlands it was Bokolma and Mul villages in Gumine District, and in the Islands Powat, Boat and Ndranou villages (inland) and Ahus, Pitulu and Andra Islands in Manus Province. In the National Capital District, approximately one-third of enrolments were done in a upper-middle class area (Korobosea/Boroko), one-third in a middle-class area (Gerehu) and one-third-in a squatter settlement area (Nine Mile).

b Fiji, Response rate, A total of 2586 people participated in the 2011 survey (response rate 2586/4850=53.3%).

Supplementary Table 2 provides historical information on the previous survey years and the characteristics of past surveys.

The central comparative summary dataset available in Mendeley Data presents the latest WHO STEPS indicators for PNG and the three neighboring Melanesian countries, including fruit and vegetable consumption, physical activity, body mass index, overweight, obesity, and waist circumference.

Supplementary Table 3 provides additional details regarding physical activity levels in PNG, stratified by sex (overall, men, and women) based on the WHO STEPS survey. The results show that 9.9% (±3.9) of the population reported low physical activity, 16.6% (±7.8) moderate, and 73.4% (±8.6) high. Among men, 9.0% (±3.7) reported low, 14.9% (±8.2) moderate, and 76.1% (±9.3) high physical activity, with the lowest levels observed in the 55–64 age group. For women, 10.9% (±4.5) reported low, 18.5% (±6.9) moderate, and 70.6% (±7.4) high physical activity, with the lowest activity levels also found in the 55–64 age group. The data used to summarize the WHO STEPS survey findings were obtained from publicly available sources (open data available at https://www.who.int/teams/noncommunicable-diseases/surveillance/data).

### 3.3 Additional health and socioeconomic surveys in PNG

Table 3 summarizes the Papua New Guinea Household Income and Expenditure Survey (PNGHIES) conducted between 2009 and 2010 [7]. The information presented in the table was collected through self-reported responses. The survey covered various domains, including family demography, education, housing conditions, ownership of durable household goods, health, employment, loans and private transfers, disputes, and personal security.

**Table 3:** Summary of the papua new guinea household income and expenditure survey (PNGHIES) 2009–2010.

Survey Items	Measurement
Family Demography	Sex
	Age group
	Type of household
	Household size
	Marital status
	Orphans among children
Education	Highest education
	Literacy level
	School enrolment
	Reasons for attending school
	Population never attended school
	Reasons for stopping to attend school
Dwelling	Characteristics of dwelling
	Access to dwelling related facilities
	Access to dwelling related services
Ownership of durable household goods	Ownership of durable household goods
	Ownership of durable goods
Health	Use of mosquito nets
	Incident of malaria
	Population reporting health complaint
	Incidences of diarrhea under six years
	Use of health care facilities
	Medicine prescribed without prescription (in the last month)
	Payment of medicine (in the last month)
	Population hospitalized
	Consumption of tobacco, betelnut and alcohol
	Prevalence of stunting, underweight, and wasting
	Disabilities
Employment	Employment status
	Unemployment (last 7 days)
	Employment type
	Profile of wage employees
	Profile of informal sector
	Profile of casual labor
	Profile of non-agriculture business
	Profile of agriculture related business
Loans and Private Transfer	Receipt of private transfer (cash or hand)
	Loan incidences
	Average loan size
Disputes	Incidences of various disputes
	Adversaries in disputes
	Property damaged caused by disputes
	Injury caused by disputes
	Deaths caused by disputes
	Dispute resolution
Personal Security	Assessment
	Issues

The survey information in the table was collected from a self-report. The measurements were categorized into sex, age group, rural, and urban.

Table 4 provides an overview of the Papua New Guinea Demographic Health Survey (PNGDHS) conducted between 2016 and 2018 [8]. The survey included women’s questionnaires focusing on children’s health and nutrition, as well as women’s health and nutrition, with no data collected on men’s health. The household questionnaire gathered health and nutritional information for both children and adults. All data were collected through self-reported responses.

**Table 4:** Overview of the papua new guinea demographic health survey (PNGDHS) (2016–2018).

Survey Items	Target category	Measurements
Women’s Questionnaires	Children’s health and nutrition information	Family planning
		Antenatal care
		Breast feeding and infant feeding practice
		Vaccination and childhood illnesses
		Vitamin A and deworming
		Micro nutrients intake
		Child clinical information
		Acute Respiratory Infections
		Fever
		Diarrhea
	Women’s health and nutrition	Smoking and chewing habits
		Maternal health care
		Clinical information
		Iron deficiency and deworming
		Salt iodization
		Vitamin and micronutrient
Household Questionnaire	Children’s health and nutrition information	Food insecurity
		Fortified nutrition
		Water and sanitation
		Wealth
	Adults’ health and nutrition information	Smoke exposures
		Household population or composition
		Parental survival (children)

The survey information in the table was collected from a self-report.

These datasets are not used for direct comparison with NNS or STEPS data but are included to document additional national surveys relevant to health and nutrition monitoring in PNG.

## 4 Experimental Design, Materials and Methods

### 4.1 Data identification and screening of NNS in PNG and three melanesian countries

National survey data were identified through a structured search conducted by two co-authors (S.S. Pau and V. Wari). The search targeted publicly available documents released by each Melanesian country, including:

• official government web pages,
• published National Nutrition Survey (NNS) reports,
• statistical office publications, and
• publicly accessible PDF documents and technical reports.

The search covered Papua New Guinea, the Solomon Islands, Vanuatu, and Fiji. For each country, the most recent national survey that included nutrition, anthropometry, or lifestyle-related indicators was identified and extracted. Stewart Sarieng Pau led the identification of survey materials for Papua New Guinea and assisted in retrieving historical survey documentation for the other three countries. All extracted sources were independently verified by co-author Vicky Wari to ensure completeness and consistency.

To ensure systematic identification, we included documents only if they:

(1) were issued by a national government ministry, statistical office, or WHO;
(2) contained aggregated results from a national-level survey; and.
(3) reported on topics related to nutrition, anthropometry, or lifestyle/physical activity.

Documents were excluded if they consisted solely of project summaries, regional sub-national reports, or publications lacking methodological description.

### 4.2 Data extraction and organization

From each national report, the following aggregated information was extracted manually:

• nutrition indicators (e.g., stunting, wasting, underweight, micronutrient status),
• anthropometric measures,
• survey methodology (sampling frame, population coverage, and historical frequency), and
• data availability (statistical summaries and access to individual-level data, if applicable).

Only publicly accessible, anonymized, aggregated information was used; no individual-level data were accessed.

All extracted information was organized into a unified comparative table structure to describe survey characteristics and key indicators across countries. Because survey designs differed across years and nations, variables were grouped into common domains through harmonization of terminology, units of measurement, and indicator categories.

Because only aggregated tabulations were available, harmonization did not involve statistical adjustment or recoding of individual-level variables. Instead, harmonization consisted of aligning definitions, indicator labels, units, and age/sex stratifications to create a consistent descriptive framework across countries.

Variables were retained only if they appeared in at least one other Melanesian country or corresponded to internationally recognized indicators (e.g., stunting, wasting, BMI categories, STEPS physical activity metrics). This ensured comparability while still preserving country-specific characteristics where appropriate.

### 4.3 Analyses of physical activity in the WHO steps survey

The data used in this section were retrieved by the authors from the official WHO STEPS website. No primary data collection was conducted; only publicly available aggregated indicators were used.

According to the 2007–2008 STEPS Fact Sheet for Papua New Guinea [6], the three physical activity domains were initially examined separately to determine the proportion of activity undertaken in each domain as part of total physical activity, as well as the mean/median minutes spent in each domain.

For all domains, three levels of activity were established: low activity, moderate activity, and high activity. To categorize participants, the total time spent on each activity per week was calculated by multiplying the number of days by the duration of the activity and summing the results across all domains. To account for the varying levels of energy expenditure (i.e., moderate or vigorous activity), the daily duration of activity was converted into MET-minutes per day. The term MET (metabolic equivalent) is used to indicate the intensity of physical activity. MET is the ratio of the metabolic rate associated with a specific activity to the resting metabolic rate. For example, the energy cost of sitting is equivalent to a resting metabolic rate of 1 MET.

In this report, the following MET values were allocated to the three physical activity domains:

• Moderate physical activity (work and leisure domain) = 4.0 METs.
• Vigorous physical activity (work and leisure domain) = 8.0 METs.
• Travel-related walking/cycling = 4.0 METs.

The following levels of activity, in terms of MET-minutes, were defined as:

• Low active: <600 MET-minutes per week.
• Moderately active: 600–1500 MET-minutes per week.
• Highly active: >1500 MET-minutes per week.

It should be noted that the activity level classifications based on MET-minutes described above are specific to the STEPS survey for PNG, and the definitions are not consistent with those used in other countries’ implementations of the STEPS methodology.

## Limitations

The primary limitation of this study is the infrequency and irregularity of national health surveys conducted in PNG and other Melanesian countries. The most recent PNG National Nutrition Survey (NNS) was conducted in 2005, following a 15-year gap, and has not been updated since. Similarly, the WHO STEPS survey was conducted only once, between 2007 and 2008. This irregularity in data collection is especially concerning, given the serious public health challenges faced by PNG, such as malnutrition, with nearly 50% of children under five being stunted, and a rising obesity rate among adults [9]. The lack of regular data collection hinders the ability to monitor long-term health trends and to address region-specific issues, such as the double burden of malnutrition and non-communicable diseases.

To ensure the reliability and applicability of health data, it is crucial to increase the frequency of such surveys in PNG, ideally adopting a routine annual schedule, as seen in other countries such as Japan. Japan’s National Health and Nutrition Survey, conducted annually since 1945 (formerly known as the National Nutrition Survey), provides comprehensive data on the population’s health, nutritional intake, and lifestyle habits [10,11]. This regular survey serves as an essential foundation for the formulation of national health policies and public health interventions [12]. A similar model could help PNG strengthen its health monitoring systems and enable prompt interventions and evidence-based public health strategies tailored to the population’s needs. This would be particularly important for addressing the urgent health challenges related to malnutrition and obesity in PNG.

## Ethics Statement

This study was conducted in accordance with the ethical guidelines for publication in Data in Brief. The data were obtained from publicly available aggregated reports accessible through the official websites of National Statistical Offices (NSOs), as well as from reputable sources such as Google Scholar, PubMed, the World Bank, UNICEF, the World Health Organization (WHO), the Food and Agriculture Organization (FAO), national Ministries of Health, the United Nations Population Fund (UNFPA), and the Centers for Disease Control and Prevention (CDC). As the study relied exclusively on anonymized annual reports and summary tables that did not include any personally identifiable information, ethical approval from the National Institutes of Biomedical Innovation, Health and Nutrition was not required.

## Credit Author Statement

**Takashi Nakagata:** Conceptualization, Investigation, Visualization, Writing – original draft, Writing – review & editing; **Stewart Sarieng PAU:** Conceptualization, Investigation, Writing – original draft, Writing – review & editing; **Vicky Wari:** Investigation, Writing – review & editing; **Masayo Rossignoli Nakamori:** Conceptualization, Investigation, Writing – review & editing, Supervision; **Rei Ono:** Conceptualization, Writing – review & editing, Supervision; **Miwa Yamaguchi:** Conceptualization, Writing – review & editing, Supervision, Project administration;

## Declaration of Generative AI and AI-Assisted Technologies in the Writing Process

During the preparation of this work, the author(s) used ChatGPT (OpenAI) to improve the grammar, phrasing, and readability of the English text, and to assist in creating reproducible R scripts for analysis and data visualization. After using this tool, the author(s) carefully reviewed and edited the content as needed and take(s) full responsibility for the content of the published article.

## Appendix Supplementary Materials

Image, application 1
