# Supplementary material for: National Nutrition and Physical Activity Surveillance in Papua New Guinea: data showing comparison with three Melanesian countries
Source: Data Brief. 2026 Aug 12;68:113170. doi: 10.1016/j.dib.2026.113170 (PMC13544349; doi:10.1016/j.dib.2026.113170)
Supplement: Supplementary file 1 [file mmc1.docx]

**Supplementary Table1.** Basic geographic, demographic, and economic characteristics of four Melanesian countries

| Countries  (Race) | Landmass (in Sq. km) ^a^ | Population (year) ^a^ | Life expectancy at birth, total (years) ^a^ | Literacy rate, adult total (%)  (years) | Gross Domestic Product (GDP) by World Bank | | | |
| --- | --- | --- | --- | --- | --- | --- | --- | --- |
|  |  |  |  |  | GDP USD In Billion (years) ^a^ | GDP Annual Growth Rate in % (years) ^a^ | GDP Per Capita In USD (years) ^a^ | GDP Per Capita Purchasing Power Parity (PPP) in USD (years) ^a^ |
| Global | - | 8.14 billion  (2023) | 73  (2023) | 87  (2023) | 111.33 trillion  (2024) | 2.9  (2024) | 13673.0 (2024) | 22,850.4  (years) |
| Melanesian |  |  |  |  |  |  |  |  |
| Papua New Guinea | 452,860 | 10,576,502  (2024) | 66  (2023) | 63.4 ^b^  (2023) | 32.54  (2024) | 4.1  (2024) | 3,076.5  (2024) | 4,633.0  (2023) |
| Solomon Islands | 27,990 | 819,198  (2024) | 71  (2023) | 76.6 ^c^  (2024) | 1.76  (2024) | 2.5  (2024) | 2,149.4  (2024) | 2,812.4  (2023) |
| Vanuatu | 12,190 | 327,777  (2024) | 71  (2023) | 89 ^a^  (2021) | 1.16  (2024) | 4.0  (2024) | 3,542.8  (2024) | 3,460.9  (2022) |
| Fiji | 18,270 | 928,784  (2024) | 67  (2023) | 99.1 ^d^  (2018) | 5.84  (2024) | 3.8  (2024) | 6288.4  (2024) | 15,152.1  (2023) |

a World Bank, <https://data.worldbank.org/>

b Asia Pacific Report, <https://asiapacificreport.nz/2023/08/08/pngs-literacy-rate-lowest-in-pacific-but-government-plans-boost-to-70/?utm_source=chatgpt.com>

c UNESCO, <https://countryeconomy.com/demography/literacy-rate/solomon-islands?utm_source=chatgpt.com>

d UNESCO, <https://www.indexmundi.com/fiji/literacy.html?utm_source=chatgpt.com>

**Supplementary Table 2.** Outline of the WHO STEPS survey for Papua New Guinea (PNG) compared with three Melanesian neighboring countries in the Oceania region—including past survey years

| Country | PNG | Solomon | | Vanuatu | | Fiji | |
| --- | --- | --- | --- | --- | --- | --- | --- |
| Year | 2007-2008 | 2006 | 2015 | 2005 | 2011 | 2002 | 2011 |
| Geographic coverage | Four regions of PNG and Port Moresby (capital only) | Honiara, Western Province and Malaita ^a^ | National coverage | Efate island only | National coverage | National coverage | National coverage |
| Universe | Adults aged 15-64 years. | Adults aged 15-64 years. | Adults aged 18-69 years. | Adults aged 15-60 years. | Adults aged 25-64 years. | Adults aged 15-64 years. | Adults aged 25-64 years. |
| Sampling procedure | The data are based on a representative population-wide sample of Papua New Guineans aged 15-64 years.  Representative areas in the four regions of PNG, as well as in the capital Port Moresby | A multi-stage cluster sample design was used to produce representative data for that age range in Solomon Islands. | A multi-stage A multi-stage cluster sample design was used to produce representative data. | Unknown | A multi-stage cluster sampling design was used to produce representative data for that age range in Vanuatu. | A multi-stage cluster sampling methodology, the targeted sample size was 7050. | A multi cluster stage sample design to produce representative data for adults aged 25-64 years. |
| Response rate | 80.2% | 94.4% | 58.4% | Unknown | 94% | Unknown ^c^ | 53.3% ^b^ |
| Sample size | A total of 2,944 individuals aged 15-64 yrs. | A total of 2,833 individuals aged 15-64 yrs, | A total of 2,525 individuals aged 18-69 yrs. | Unknown | A total of 4,649 individuals aged 25-64 yrs. | A total of 6,783 individuals aged 15-64 yrs. | A total of 2,586 individuals aged 25-64 yrs. |
| Dietary intake measurements | Dietary assessments were made by brief dietary assessment instruments: a retrospective methods in which a limited number of specified food and drinks (Fruits and vegetables) were usually consumed. Mean number of days they were consumed in a typical week and mean number of servings on average per day was asked as a semi-quantitative instrument. The questionnaire was interview-administered (face-to-face). | Dietary assessments were made by brief dietary assessment instruments: a retrospective methods in which a limited number of specified food and drinks (Fruits and vegetables) were usually consumed. Mean number of days they were consumed in a typical week and mean number of servings on average per day was asked as a semi-quantitative instrument. The questionnaire was interview-administered (face-to-face). | Food items were added for rice and noodles, bread and bakery products, and sugar-added drinks. For dietary salt, respondents were asked about the amount and frequency of salt or salty sauces added, the amount of salty processed food consumed, their knowledge of salt and its impact on health, and the actions they have taken to control salt intake. For oil and fat used in meal preparation, respondents wee additionally asked about the frequency of eating out and the frequency of meals containing coconut, cream or lolo. | Not available | Dietary assessments were made by brief dietary assessment instruments: a retrospective methods in which a limited number of specified food (Fruits and vegetables) were usually consumed. Mean number of days they were consumed in a typical week and mean number of servings on average per day was asked as a semi-quantitative instrument. The questionnaire was interview-administered (face-to-face) | Dietary assessments were made by brief dietary assessment instruments: a retrospective methods in which a limited number of specified food (Fruits and vegetables) were usually consumed. Mean number of days they were consumed in a typical week and mean number of servings on average per day was asked as a semi-quantitative instrument. The questionnaire was interview-administered (face-to-face).  In addition, the type of oil or fat used in food preparation at household was asked. | The same as the survey conducted in 2002. |
| Physical activity measurements | Survey participants were asked how often (frequency) and how long (duration) they engaged in three domains of physical activity: during recreation or leisure time, work and transport in a typical week. In the work and leisure domains, respondents were asked how many days per week and how many hours/minutes per day they participate in moderate- and vigorous-intensity activities. In the transport domain, respondents were asked how often and how long they either walk and/or cycle to and from places. | Respondents were asked how often (frequency) and how long (duration) they engaged in three domains of physical activity in a typical week: work-related, transport-related and leisure-related. In the work and leisure domains, respondents were asked how many days per week and how many hours/minutes per day they participate in moderate and vigorous intensity activities. In the transport domain, respondents were asked how often and how long they either walk and/or cycle to and from places. | A population’s physical activity (or inactivity) can be described in different ways. The two most common ways used for analyzing Global Physical Activity Questionnaire (GPAQ) data are:  1) To estimate a population’s mean or median physical activity using a continuous indicator such as MET-minutes per week or time spent in physical activity.  2) To classify the population into specific groups by setting cut-off points for a specific amount of physical activity. | Unknown | Respondents were asked how often (frequency) and how long (duration) they engaged in three domains of physical activity in a typical week: work-related, transport-related and leisure-related. In the work and leisure domains, respondents were asked how many days per week and how many hours/minutes per day they participate in moderate and vigorous intensity activities. In the transport domain, respondents were asked how often and how long they either walk and/or cycle to and from places. | Physical activity participation in the Fiji STEPS survey was measured by asking participants to report on the frequency and number of different types of activity they undertook on a typical day as part of work, travel and leisure. | Unknown |

<https://extranet.who.int/ncdsmicrodata/index.php/catalog/STEPS/?page=1&ps=15&repo=STEPS>

^a^ For the Papuan sample, the villages of Lese Havihara and Lese Kavora in Gulf Province were selected as representative. For Momase, Ambana and Mambuan villages in Bogia District of Madang Province were studied. In the Highlands it was Bokolma and Mul villages in Gumine District, and in the Islands Powat, Boat and Ndranou villages (inland) and Ahus, Pitulu and Andra Islands in Manus Province. In the National Capital District, approximately one-third of enrolments were from in an upper-middle class area (Korobosea/Boroko), one-third in a middle-class area (Gerehu) and one third in a squatter settlement area (Nine Mile).

^b^ Fiji, Response rate, A total of 2,586 people participated in the 2011 survey (response rate 2,586/4,850=53.3%)

^c^ A total of 6794 adults participated in the Fiji STEPS survey.

**Supplementary Table 3**. Physical activity by age group and gender for Papua New Guinea—results of the STEPS Survey, based on publicly available data from the WHO STEPS Survey (2007–2008).

Supplementary Table 3-1. Categories of total physical activity by age groups.

| Group | Age group | Number | Low | | Moderate | | High | |
| --- | --- | --- | --- | --- | --- | --- | --- | --- |
|  |  |  | (%) | 95% CI | (%) | 95% CI | (%) | 95% CI |
| Overall | 15-24 | 714 | 9.5 | 5.2-13.8 | 14.9 | 7.5-22.4 | 75.6 | 64.9-86.3 |
|  | 25-34 | 641 | 8.1 | 3.4-12.7 | 12.6 | 2.6-22.7 | 79.3 | 68.7-89.9 |
|  | 35-44 | 556 | 6.8 | 3.2-10.3 | 16.9 | 9.7-24.1 | 76.3 | 68.1-84.5 |
|  | 45-54 | 412 | 13 | 7.0-19.0 | 20.9 | 10.3-31.6 | 66.1 | 59.4-72.8 |
|  | 55-64 | 290 | 24 | 10.5-37.5 | 33.8 | 26.0-41.6 | 42.2 | 31.9-52.6 |
|  | 15-64 | 2613 | 9.9 | 6.1-13.8 | 16.6 | 8.9-24.4 | 73.4 | 64.9-82.0 |
| Men | 15-24 | 336 | 7.2 | 4.0-10.4 | 11.8 | 1.5-22.2 | 80.9 | 69.1-92.8 |
|  | 25-34 | 313 | 10 | 2.2-17.8 | 13.3 | 3.2-23.4 | 76.7 | 61.3-92.2 |
|  | 35-44 | 269 | 7.2 | 3.8-10.7 | 13.7 | 6.2-21.2 | 79.1 | 69.2-89.0 |
|  | 45-54 | 219 | 8.5 | 4.5-12.5 | 17.3 | 6.6-28.1 | 74.1 | 65.7-82.5 |
|  | 55-64 | 161 | 19.6 | 7.5-31.7 | 34.8 | 20.9-48.6 | 45.6 | 26.2-65.0 |
|  | 15-64 | 1298 | 9 | 5.3-12.7 | 14.9 | 6.7-23.1 | 76.1 | 66.8-85.4 |
| Women | 15-24 | 378 | 11.9 | 6.8-17.0 | 18.3 | 15.0-21.5 | 69.8 | 62.0-77.7 |
|  | 25-34 | 328 | 6.1 | 2.6-9.7 | 12 | 0.9-23.1 | 81.9 | 73.5-90.2 |
|  | 35-44 | 287 | 6.3 | 1.1-11.5 | 20.2 | 13.4-27.1 | 73.5 | 66.7-80.3 |
|  | 45-54 | 193 | 18 | 9.2-26.8 | 24.9 | 12.0-37.9 | 57.1 | 48.8-65.3 |
|  | 55-64 | 129 | 29.2 | 12.5-46.0 | 32.6 | 25.6-39.6 | 38.2 | 25.2-51.1 |
|  | 15-64 | 1315 | 10.9 | 6.4-15.4 | 18.5 | 11.6-25.4 | 70.6 | 63.3-78.0 |

Supplementary Table 3-2. Mean minutes of total physical activity (mean MET minutes per day) on average per day by gender and age group.

| Age group | Overall | | | Men | | | Women | | |
| --- | --- | --- | --- | --- | --- | --- | --- | --- | --- |
|  | number | Mean minutes | 95% CI | number | Mean minutes | 95% CI | number | Mean minutes | 95% CI |
| 15-24 | 714 | 173.4 | 117.1-229.7 | 336 | 190.8 | 115.5-266.1 | 378 | 154.9 | 126.2-183.5 |
| 25-34 | 641 | 205 | 133.5-276.5 | 313 | 222.8 | 116.1-329.4 | 328 | 187.3 | 158.4-216.2 |
| 35-44 | 556 | 198.4 | 162.6-234.1 | 269 | 212.8 | 152.3-273.4 | 287 | 183.5 | 171.7-195.4 |
| 45-54 | 412 | 172.7 | 144.1-201.2 | 219 | 180.6 | 162.0-199.2 | 193 | 163.7 | 111.2-216.2 |
| 55-64 | 290 | 118.7 | 104.5-132.8 | 161 | 120.8 | 101.2-140.3 | 129 | 116.1 | 88.2-144.1 |
| 15-64 | 2613 | 183.5 | 133.5-233.6 | 1298 | 197.7 | 129.1-266.4 | 1315 | 168.6 | 141.7-195.4 |

Supplementary Table 3-3. Mean minutes of work-related physical activity (mean MET minutes per day) by gender and age group.

| Age group | Overall | | | Men | | | Women | | |
| --- | --- | --- | --- | --- | --- | --- | --- | --- | --- |
|  | number | Mean minutes | 95% CI | number | Mean minutes | 95% CI | number | Mean minutes | 95% CI |
| 15-24 | 714 | 92.9 | 70.9-114.9 | 336 | 98.9 | 68.4-129.4 | 378 | 86.5 | 74.6-98.4 |
| 25-34 | 641 | 127.5 | 88.6-166.5 | 313 | 133.4 | 83.7-183.1 | 328 | 121.7 | 95.6-147.7 |
| 35-44 | 556 | 127.5 | 118.4-136.6 | 269 | 138.6 | 114.4-162.6 | 287 | 116.1 | 98.1-134.2 |
| 45-54 | 412 | 102 | 92.7-111.2 | 219 | 111 | 92.0-130.0 | 193 | 91.9 | 79.2-104.6 |
| 55-64 | 290 | 67.2 | 47.7-86.7 | 161 | 66.1 | 35.6-96.6 | 129 | 68.5 | 54.6-82.4 |
| 15-64 | 2613 | 108.8 | 90.2-127.5 | 1298 | 115.2 | 89.4-141.1 | 1315 | 102.1 | 91.5-112.7 |

Supplementary Table 3-4. Mean minutes of transport-related physical activity (mean MET minutes per day) by gender and age group.

| Age group | Overall | | | Men | | | Women | | |
| --- | --- | --- | --- | --- | --- | --- | --- | --- | --- |
|  | number | Mean minutes | 95% CI | number | Mean minutes | 95% CI | number | Mean minutes | 95% CI |
| 15-24 | 714 | 60.6 | 34.6-86.6 | 336 | 67.1 | 29.1-105.2 | 378 | 53.6 | 42.7-64.4 |
| 25-34 | 641 | 63.2 | 42.1-84.2 | 313 | 69.8 | 28.5-111.1 | 328 | 56.5 | 51.3-61.7 |
| 35-44 | 556 | 64.9 | 39.4-90.4 | 269 | 68.5 | 33.3-103.8 | 287 | 61.1 | 46.2-76.0 |
| 45-54 | 412 | 66 | 36.6-95.4 | 219 | 66.1 | 40.1-92.0 | 193 | 66 | 32.5-99.5 |
| 55-64 | 290 | 49.7 | 32.8-66.6 | 161 | 53.2 | 41.9-64.5 | 129 | 45.5 | 23.0-68.1 |
| 15-64 | 2613 | 62.1 | 37.9-86.3 | 1298 | 67 | 31.9-102.1 | 1315 | 56.8 | 45.3-68.4 |

Supplementary Table 3-5. Mean minutes of recreation-related physical activity (mean MET minutes per day) by gender and age group.

| Age group | Overall | | | Men | | | Women | | |
| --- | --- | --- | --- | --- | --- | --- | --- | --- | --- |
|  | number | Mean minutes | 95% CI | number | Mean minutes | 95% CI | number | Mean minutes | 95% CI |
| 15-24 | 714 | 20 | 10.0-30.0 | 336 | 24.8 | 16.0-33.6 | 378 | 14.8 | 5.9-23.6 |
| 25-34 | 641 | 14.3 | 1.9-26.8 | 313 | 19.6 | 2.6-36.6 | 328 | 9.1 | 3.1-15.0 |
| 35-44 | 556 | 6 | 0.8-11.2 | 269 | 5.7 | 2.5-8.9 | 287 | 6.3 | 0.0-12.9 |
| 45-54 | 412 | 4.6 | 3.1-6.1 | 219 | 3.6 | 0.0-8.7 | 193 | 5.8 | 0.0-12.9 |
| 55-64 | 290 | 1.8 | 1.5-2.1 | 161 | 1.5 | 0.0-4.3 | 129 | 2.1 | 0.0-5.5 |
| 15-64 | 2613 | 12.6 | 4.4-20.8 | 1298 | 15.5 | 6.3-24.6 | 1315 | 9.6 | 3.2-16.1 |
